# The Interplay of Curvature, Geometry, and Topology Shapes Tissue Organisation in Epithelial Shells

**DOI:** 10.1002/advs.77487

**Published:** 2026-09-28

**Authors:** Laura Morato, Jesús A. Andrés‐San Román, Juan Garrido‐García, Carmen Gordillo‐Vázquez, Ana M. Palacios, Elisabeth Lidueña‐Fernández, Katie McDole, Pedro Gómez‐Gálvez, Valentina Annese, Luis M. Escudero

**Affiliations:** ^1^ Instituto de Biomedicina de Sevilla (IBiS) Hospital Universitario Virgen del Rocío/CSIC/Universidad de Sevilla Departamento de Biología Celular Facultad de Biología Universidad de Sevilla Seville Spain; ^2^ Instituto de Biomedicina de Sevilla (IBiS) Hospital Universitario Virgen del Rocío/CSIC/Universidad de Sevilla Departamento de Matemática Aplicada I Universidad de Sevilla Seville Spain; ^3^ MRC Laboratory of Molecular Biology Cambridge UK; ^4^ Biomedical Network Research Centre on Neurodegenerative Diseases (CIBERNED) Madrid Spain

**Keywords:** curvature, epithelial shells, tissue topology, voronoi diagrams

## Abstract

During morphogenesis, multicellular patterns emerge from coordinated processes at the single‐cell level. This is characterised in flat epithelial layers, where average neighbour distributions follow conserved arrangements. However, less is known about how curvature defines tissue architecture. Epithelial shells constitute a critical structural stage in early mammalian embryonic development. Our analysis reveals how the closed, curved geometry of epithelial shells limits their 3D organisation. Here, we designed computational models that predict shell surface topology across different cell numbers. To validate the model, MDCK cysts with precise cell‐level segmentation were analysed. Our results show that more pentagons are required to accommodate higher curvature per cell patch, a restriction also observed in early mouse embryos. Small cysts and embryos exhibited a restricted subset of polygon configurations, predominantly those maximising the area‐to‐perimeter ratio. We conclude that epithelial shells are organised under general rules mirroring other natural cages, revealing that geometric and curvature constraints guide early epithelial organisation.

## Graphical abstract

Purely geometric 3D Voronoi models predict the organisation of epithelial shells. The interplay of curvature, geometry, and topology enriches the number of pentagons in shells with lower number of cells. Small MDCK cysts and Mouse embryos match the top polygonal configurations in a compactness‐based ranking derived from the Voronoi model. In conclusion, shell organisation, while diverse, is fundamentally non‐arbitrary.

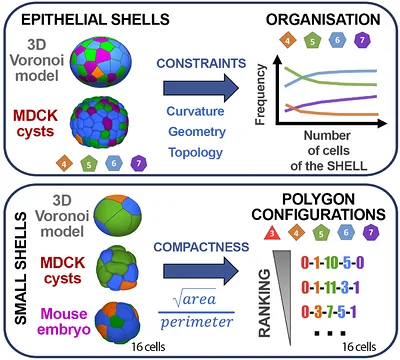

## 1 Introduction

In animals, the organisation of cells into epithelial sheets is an essential feature from the developing embryo to the morphogenesis of complex organs such as liver, lungs or kidneys [1, 2]. The characteristic polygonal tessellation of epithelial cells has been presumed to reflect the influence of optimal cell packing in 2D [3]. Over two decades ago, seminal studies proposed that the cell division process was sufficient to explain the predominance of hexagonal cells as well as the overall polygonal distribution of the tissue [3, 4, 5]. Subsequent research indicated that epithelia are governed by physical constraints that drive self‐organisation in two dimensions not only dependent on proliferation [6]. The geometrical and organisational characteristics of 2D epithelial sheets have been successfully simulated using mathematical constructs such as Voronoi diagrams [6, 7, 8]. These models reproduce the polygon distributions observed across diverse epithelial tissues based on the variance of the cell area distributions: the frequency of hexagons increases when the cell area values are more homogeneous. Importantly, the Voronoi algorithm also recapitulates the tissue's underlying energy‐minimising mechanisms in both 2D surfaces and 3D architecture of epithelial tissues [6, 9, 10, 11]. Furthermore, the Voronoi framework provides the geometric foundation for more complex mechanical models. For instance, it represents the energetic ground state of a Vertex Model under uniform interfacial tension without requiring unknown biophysical parameters [9]. This framework serves to isolate how geometry and curvature alone dictate tissue architecture.

Although informative, the 2D study of epithelial tissues fails to capture important features of tissue organisation. It has become clear that there is a need to analyse the 3D morphology of epithelial cells to study organogenesis [12, 13, 14], cell migration [15], branching formation [16], tumourigenesis [17] or wound healing [18]. A paradigm of the importance of the 3D analyses, is the description that epithelial cells can adopt a novel geometrical shape known as scutoid [11]. Scutoids have been shown to have a role in morphogenesis as well as in the biophysical properties of tissues, allowing different neighbouring cells in apical and basal surfaces [19], cushioning and minimising cell surface tension and leading to a balanced energetic state [10, 11, 20, 21, 22, 23, 24]. Scutoid formation is influenced by factors such as cell density [25] and steep tissue curvature [26], and also enables the transition from monolayer to stratified epithelium [27].

In addition to sheets and tubes, epithelia can also form closed spheroidal assemblies. Epithelial shells are a widespread structure, functionally important during embryogenesis. In this context, certain epithelial systems including cysts, organoids, and mammalian embryos, possess the capability to self‐organise forming structures with a lumen (Figure 1A–E) [28, 29, 30, 31]. Closed shells also appear in non‐biological systems, with a geometric order due to a tendency either to minimise surface energy or maximise space filling [32, 33, 34, 35]. This is the case for fullerenes, a large family of carbon allotropes whose atoms form a closed cage, defining a hollow surface of interconnected hexagons and pentagons (Figure 1F) [36, 37]. The simplest case takes the form of a dodecahedron, where twelve pentagons can enclose an inner space (Figure 1F). Thus, fullerenes can serve as a natural benchmark to understand how geometric and topological constraints dictate the distribution of the polygonal faces in closed‐cage structures.

**FIGURE 1:**
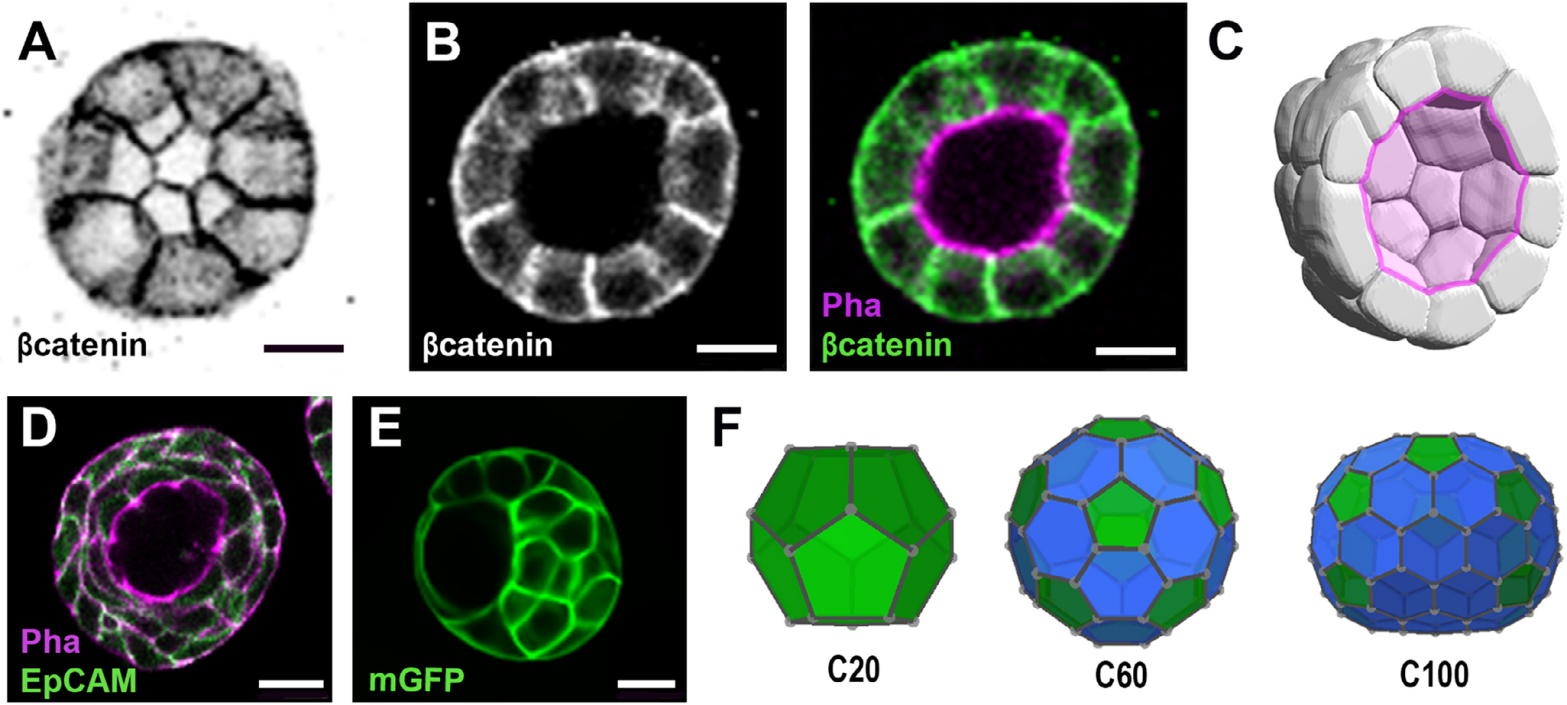
Epithelial and other natural shells. (A) Maximum Z‐projection of half a MDCK cyst after 4 days of culture showing the outlines of the cells immunolabelled for β‐catenin protein. Scale bar, 10 µm. (B) Confocal section of the equatorial plane of the same cyst showing the lumen outlined by phalloidin (magenta, apical surface of cells). (C) 3D reconstruction of a half cyst to show the arrangement of the cells around the lumen. The pink region marks the outline of the lumen. (D) Representative confocal image of a cross section of human lung organoid stained with phalloidin (magenta) and immunolabelled for the EpCAM cell surface marker (green). Scale bar, 15 µm. (E) Confocal image extracted from a movie of a representative mouse embryo at the early blastocyst stage, ubiquitously expressing a membrane‐GFP transgene. Scale bar, 20 µm. (F) Examples of fullerene carbon molecules: dodecahedral fullerene (C20), buckminsterfullerene (C60), and C100 fullerene. The vertices represent carbon atoms forming shells composed of pentagons (green) and hexagons (blue).

Epithelial shells can be topologically regarded as convex polyhedra, in which individual cells behave as polygonal faces that share edges pairwise and meet at tricellular junctions. These geometrical restrictions confer the topological property of an ideal trivalent network, which implies that it must satisfy a condition derived from *Euler's theorem*: the total number of edges, *E*, scales as *E = 3n‐6*, where *n* is the number of cells (faces) [38, 39]. A second constraint of epithelial shells is related with curvature: studies in 3D topology have demonstrated that the equilibrium states of closed shells are governed by the necessity of pentagons, since the internal angles of hexagons are too large to curve and close the surface of a polyhedral structure by themselves [32, 40, 41, 42]. Therefore, the surface organisation of epithelial shells is subject to fundamental topological constraints that are absent in planar epithelia. Consistent with this, theoretical modelling of cellular connectivity predicts that sufficiently large epithelial cysts (more than 13 cells) minimise their energy by adopting a “soccer‐ball” configuration at mechanical equilibrium [43]. This relationship between energy minimisation and topology has also been described in early mammalian embryos [44], where, despite initial stochasticity, cell packing converges toward a limited set of robust 3D configurations.

While multicellular patterning in flat epithelial layers is well‐characterised, the impact of both curvature, geometrical and topological constraints on tissue organisation within closed‐shells remains largely unexplored. In this study, we employ a 3D Voronoi model of epithelial spheroids to predict the topology of hundreds of MDCK‐II (Madin–Darby canine kidney) cysts in a range of size from 8 to 123 cells. The Voronoi approach allows us to bypass complex mechanical modelling and isolate the fundamental geometric and topological principles governing organisation on closed curved surfaces.

Here, we combine the analysis of a large 3D experimental dataset with a simple geometric model to show that epithelial shell organisation, while diverse, is fundamentally non‐arbitrary and driven by simple structural constraints rather than complex mechanical forces. We demonstrate that the interplay of curvature, geometry, and topology dictates tissue architecture across structurally distinct systems, including hollow cysts and filled mouse embryos. By evaluating these principles under various altered biophysical conditions, such as low‐serum culture and ROCK inhibition, we confirm that tissue organisation remains robust despite these structural perturbations. We introduce a configuration‐ranking analysis enabled by our Voronoi model framework, showing that complex polygon arrangements in small epithelial shells can be quantitatively predicted using a simple descriptor of compactness. Furthermore, we show that the presence of scutoids in epithelial cysts enables transitions between low‐ and high‐ranked configurations from apical to basal surfaces, promoting more compact basal packing (outer surface). Together, our findings establish a clear, testable framework demonstrating that the constraints of shaping a closed‐surface tessellation govern epithelial organisation independently of tissue‐specific or complex mechanical cues.

## 2 Results

### 2.1 A Curvature‐Based Voronoi Model Explains Cell Packing in 3D Epithelial Shells

In natural closed cages such as fullerenes, the number of total elements influences the polygon distribution: as the number of components increases, hexagons become relatively more abundant than pentagons [36] (Figure 1F). Therefore, if epithelial shells are under similar constraints, the number of cells should affect their organisation. According to the Gauss‐Bonnet theorem [45], increasing the number of surface patches covering a spheroidal surface results in a decrease in the average Gaussian curvature per patch. To test how the number of epithelial cells affect the organisation under the closed‐shell condition, we designed a mathematical model that reproduces key features of these structures: (1) 3D surfaces of cells are built using a geodesic Voronoi algorithm on a spheroid surface (Figure 2A,B, and Figure S1; Methods) (2) We mimicked epithelial cell arrangement by distributing Voronoi seeds across the surface to generate each cell. Starting from a random distribution of Voronoi cells (‘Voronoi 1’), we used Lloyd's algorithm to space their seeds more evenly. This method repeatedly moves each seed to the centroid of its cell to make the distances between them more uniform. We applied five iterations (‘Voronoi 6’) to reproduce a natural tissue organisation (Figure 2B and Figure S1A; see details on Methods) [6, 46]. (3) From each Voronoi seed, a 3D cell can be generated between two closed surfaces after cell interpolation along the apico‐basal axis. Therefore, it is possible to analyse different cell heights and lumen sizes (Figure 2B). (4) The 3D structure of the shell can be generated with a variable number of cells to study the influence of the curvature per patch and adopt different sizes and shapes spanning from spheres to oblate spheroids (Figure 2C and Figure S1).

**FIGURE 2:**
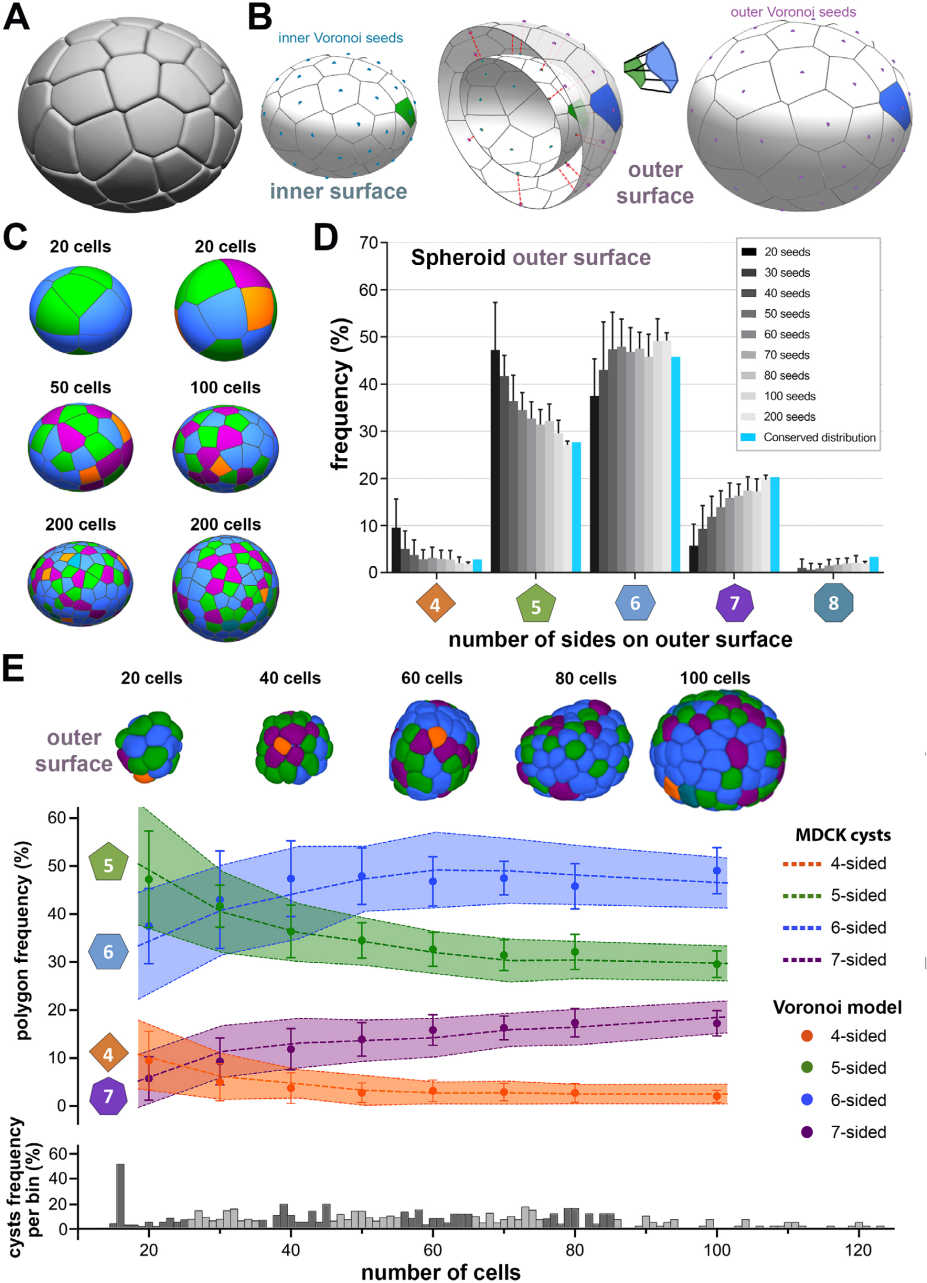
A computational model to simulate epithelial shells. (A) 3D geodesic Voronoi model imitating an epithelial shell. (B) Left, a set of seeds (light blue dots) are placed on the surface of a spheroid to build a Voronoi diagram. This corresponds to the inner surface of the model. Centre, the seeds from the inner surface are projected onto the outer surface (violet dots). This projection occurs along the normal vector to the inner surface for each seed (dotted red lines). Right, the complete model is built. The model allows for neighbour changes along the apico‐basal axis. As an example, the panel shows a scheme where a cell with five neighbours on the inner surface (green surface) can contact six cells on the outer surface (blue surface). (C) Examples of models with different shapes and number of cells; from left to right and top to bottom: 20 cells oblate spheroid, 20 cells sphere, 50 cells oblate spheroid, 100 cells oblate spheroid, 200 cells oblate spheroid, 200 cells sphere. In all figures, the polygon type is indicated by colour: quadrilateral, orange; pentagonal, green; hexagonal, blue; heptagonal, purple; octagonal, dark turquoise. (D) Average polygon distribution of the outer surface of a series of models (oblate spheroid) with increasing number of cells (N = 20 samples for each number of seeds). Models with fewer cells show the highest percentage of pentagons. As the number of cells increases, the arrangements become more similar to the conserved polygon distribution of planar epithelia (data from ref. [3]). Data show the average polygon distribution, with error bars indicating the standard deviation (s.d). (E) Top: Segmentation of MDCK cysts with increasing number of cells. Middle: The Voronoi model predicts the average distribution of polygons on the outer surface of MDCK cysts at different sizes. To show the tendency of the polygon distribution, cysts are grouped into eight bins given their number of cells (see Methods). The dashed lines are a visual guide connecting the mean values of each bin for each polygonal class. Shaded areas represent the s.d. Coloured dots indicate the average values of each polygon class for Voronoi simulations, with error bars representing the corresponding s.d. There are no significant differences between both sets of data by Wilcoxon paired test. Spearman's correlation coefficient: 0.98. Bottom: The distribution of cysts frequency per number of cells within each bin (alternatively labelled in dark or light grey).

As a first step to measure surface organisation, we calculated their average distribution of neighbours on their inner and outer surfaces (Figure 2D and Figure S1; Methods). Additionally, to assess how curvature scales with cell number (N), we established a Gauss–Bonnet theoretical baseline [45]: partitioning the total Gaussian curvature of a closed shell (4π) by N, provides a proxy of the expected curvature per cell patch (4π/N) (Figure S1B, right axis; Methods). This inverse scaling was reproduced by the cell‐to‐shell surface area ratio, which showed minimal cell area variation across the shell (Figure S1B, left axis) and, together with direct measurements, validated the used baseline (Figure S1C; Methods). Consistently, the model predicted that as the number of cells increases, the average cell‐patch curvature and the proportion of four‐ and five‐sided cells on the outer surface decrease, leading to a pattern dominated by hexagons, similar to the “conserved polygon distribution” described for planar surfaces (Figure 2D, Figure S1B,C) [3, 6]. We observed a similar trend when analysing the inner surface of the 3D shells, and it persisted in spheres (Figure S1D,E; Methods; see also Table S1). To validate whether the results obtained by the computational model recapitulate the 3D distribution of neighbours in vivo, we imaged and realistically segmented MDCK cysts of a large range of sizes cultured for 3, 4, 7, or 10 days (Figure 2E; Methods). We used CartoCell software [29] for their segmentation and to identify the neighbours of every cell on both surfaces and along their apico‐basal axis (N = 486 cysts; Table S2). Notably, when we grouped the empirical data into bins of samples with similar cell numbers, the frequencies of each polygon class closely matched those obtained from simulations, including a higher standard deviation in the samples with fewer cells (Figure 2E and Figure S2A,B; Methods; see also Tables S1 and S2). Therefore, our Voronoi model reproduces the relationship between curvature constraints and the number of cells forming closed shells, and explains the global topology of the inner and outer surfaces of epithelial cysts, including how curvature influences the convergence toward a stable equilibrium distribution as the number of cells increases (Figure 2 and Figures S1 and S2).

### 2.2 Closed‐Shell Constraints Determine Epithelial Organisation Despite Changes in Cell Proliferation

Epithelial packing has been traditionally associated with cellular mechanical properties and tissue proliferative dynamics [3, 9, 25, 47]. To examine the effect of cell division and the rate of proliferation on the observed organisation, we cultured MDCK cysts under low‐nutrient conditions (2%FBS, Fetal Bovine Serum, Figure 3A). We chose 2%FBS concentration because cysts formation was not observed at lower concentrations (Methods). First, we verified in 2D cell cultures a prominent decrease in the proliferation rate after 6 days of culture (Figure S3A). To evaluate the impact of nutrient deprivation on cystogenesis, control cysts (cultured in 10%FBS, named Control) and those cultured in 2%FBS were analysed at the same time points: 4, 7, and 10 days of culture. We confirmed that under low nutrient conditions, cells formed cysts with the same efficiency as the Control group (Figure S3B). In total, 310 2%FBS cysts were segmented. At each time point analysed, cysts grown in 2%FBS contained fewer cells than controls, resulting in a significant reduction in both total cyst volume and lumen volume (Figure 3B and Figure S3C,D). To analyse geometric features independently of the time of culture, we pooled the data from Control and 2%FBS cysts and grouped them into four bins of samples with similar cell numbers: 20, 30, 40, and 50 cells (Figure 3C and Figure S4A–D; Methods). We found a tendency toward larger volume and greater cell height in the 2%FBS samples as the number of cells increased (Figure 3C and Figure S4A,C). Despite the geometric differences between conditions within each bin, the average polygon distributions were similar when all experimental bins from the 2%FBS condition were globally compared with both the Voronoi simulations (Figure 3D) and Control cysts (Figure 3E,F and Figure S4E–G). These results suggest that reduced proliferation rate does not affect the surface organisation of these epithelial shells. Although 2%FBS cysts exhibit a delayed growth rate compared to controls, their polygon distributions remain highly consistent within equivalent cell‐number bins. Overall, this supports the idea that closed‐shell geometry imposes strong constraints on cell packing, preserving topology despite changes in growth kinetics.

**FIGURE 3:**
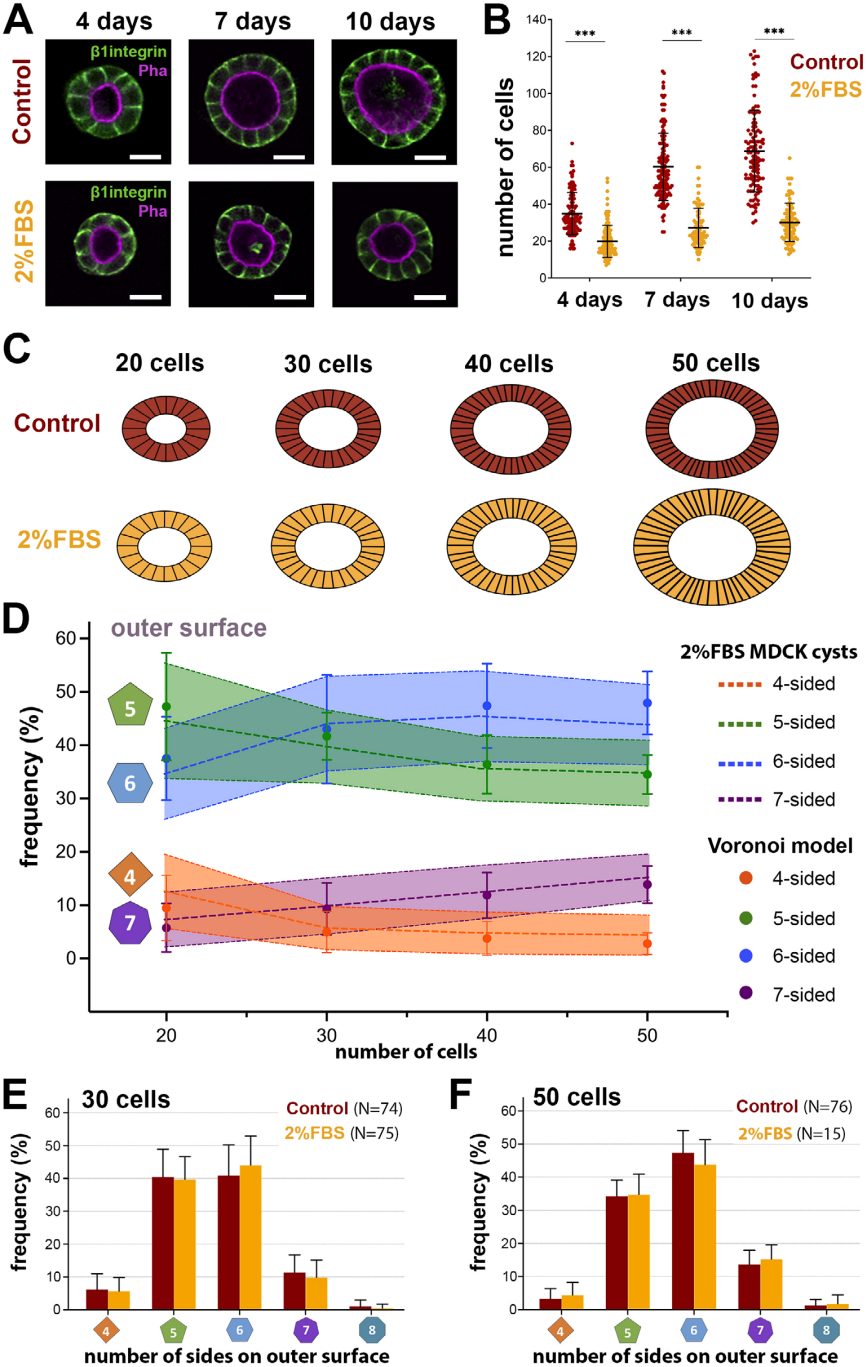
Tissue organisation under reduced proliferation. (A) Confocal sections of Control (dark red) and 2%FBS (dark yellow) representative cysts (β1‐integrin, green; phalloidin, magenta) at 4, 7, and 10 days of culture. Scale bar, 10 µm. (B) Comparison of number of cells per cyst at different days of culture: 4 days (Control, N = 119; 2%FBS, N = 137), 7 days (Control, N = 142; 2%FBS, N = 88), and 10 days (Control, N = 114; 2%FBS, N = 89). Each dot represents a single cyst. Bars indicate the mean ± s.d. ***p < 0.001 by Mann–Whitney tests with Bonferroni multiple comparisons correction. (C) Schematic representation of the geometric features of Control and 2%FBS cysts grouped by cell number (see Methods). (D) Polygon frequency at the outer surface in 2%FBS cysts within the size range of 20–50 cells closely matches the Voronoi model. There are no significant differences between both sets of data by Wilcoxon paired test. Spearman's correlation coefficient: 0.97. (E,F) Polygon distributions of Control and 2%FBS cysts for 30‐cell bin (26–35 cells) (E) and 50‐cell bin (46–55 cells) (F). Data are shown as mean + s.d. There are no significant differences between both polygon distributions at different cyst sizes by PERMANOVA.

In addition, we tested another condition that could affect cysts organisation: inhibition of myosin II activation. The actomyosin cytoskeleton influences cell shape by modulating surface tension [48, 49, 50]. Cerruti and collaborators proposed that after reaching mechanical equilibrium through a balance of proliferation rate and cortical actin relaxation, cysts acquire a topological equilibrium that resembles the “soccer ball” configuration [43]. They suggested that attenuating cortical tension through Rho kinase (ROCK) inhibition during cystogenesis could be a way to reach mechanical equilibrium and alter the topological organisation of MDCK cysts [43]. To test this, we compared a large number of both Control cysts and those treated with the ROCK inhibitor Y‐27632, that induced a strong reduction of phospho‐Myosin Light Chain II (pMLCII) (Control, N = 166; ROCK, N = 65; Figure S5; Methods) [43, 51]. To enable statistical comparisons, we organised the samples into two bins of cysts of approximately 20 and 30 cells. Importantly, we did not detect significant differences in polygon distributions compared with untreated controls, suggesting that shell organisation depends on other factors (Figure S5B). In summary, our results quantifying polygonal cell topology across a large number of samples suggests that perturbations of cortical tension can alter certain aspects of cyst geometry (such as “cell volume” or “cell height” in cysts of the 20‐cell bin and 30‐cell bin respectively; Figure S5E,F) without affecting epithelial organisation. Therefore, we interpret that cortical tension is not a major determinant of cyst organisation.

Altogether, our results support the idea that curvature plays an important role in driving the organisation of epithelial shells. We then examined the early growth stages, where curvature per cell‐patch has a stronger influence, by performing a detailed analysis of Control cysts cultured for 3 days (Figure 4 and Figure S6). Notably, we found a non‐uniform distribution of cyst frequency per number of cells in the control group. This distribution features a pronounced peak at 16 cells and a secondary peak at 8 cells (Figure 4A). These peaks could correspond to stable developmental stages captured within our narrow culture time window (3 days), following consecutive, synchronised rounds of cell division that align with complete lumen clearance. Since our dataset is composed strictly of single‐layer and single‐lumen cysts, multilayered phenotypes, which may correspond to intermediate cell numbers, are excluded from the analysis. This pattern was not observed in 2%FBS cysts within the same cell number range (4 days of culture, ensuring complete open lumen, Figure 4A; Methods). In this low‐serum condition, cell proliferation slows down and may lose synchronisation, resulting in a more continuous distribution of intermediate cell numbers. In our analysis, both the Voronoi model, Control and 2%FBS cysts with fewer cells (20‐cell bin) displayed greater variability in polygon distributions, reflecting a strong sensitivity of the topology to cell number (Figures 2E and 3D; see also Tables S1 and S2). To ensure reliable comparisons, we then restricted the analysis of topological features to samples with exactly the same number of cells. We selected 8‐cell and 16‐cell cysts from both Control and 2%FBS conditions because these were the most prevalent in the control group (Figure 4B; Methods). Sixteen‐cell cysts from both conditions presented similar tissue volume or cell volume (Figure 4C and Figure S6B). However, FBS reduction led to a significant increase in lumen size, and a decrease in cell height (Figure 4C and Figure S6A). Moreover, we did not observe significant differences in tissue organisation when comparing the oblate spheroid Voronoi model with either cyst condition (Figure 4D and Figure S6G). In all cases (8‐ and 16‐cell Voronoi model and cysts), there was a clear predominance of pentagons, around 50% (Figure 4D). For 8‐cell Control and 2%FBS cysts, the geometric changes followed a similar trend to those observed in 16‐cell cysts (Figure S6C–F). In this case, we found a major topological restriction since all the cysts and the majority of Voronoi models presented 50% of quadrilaterals and 50% of pentagons (Figure 4D and Figure S6G). Altogether, our findings demonstrate that the closed‐shell condition and cell number dictate structural rules defined by average cell‐patch curvature (Gauss–Bonnet's theorem, see Methods) and topological constraints (Euler's theorem), that keep organisation in epithelial shells robust to biological perturbations.

**FIGURE 4:**
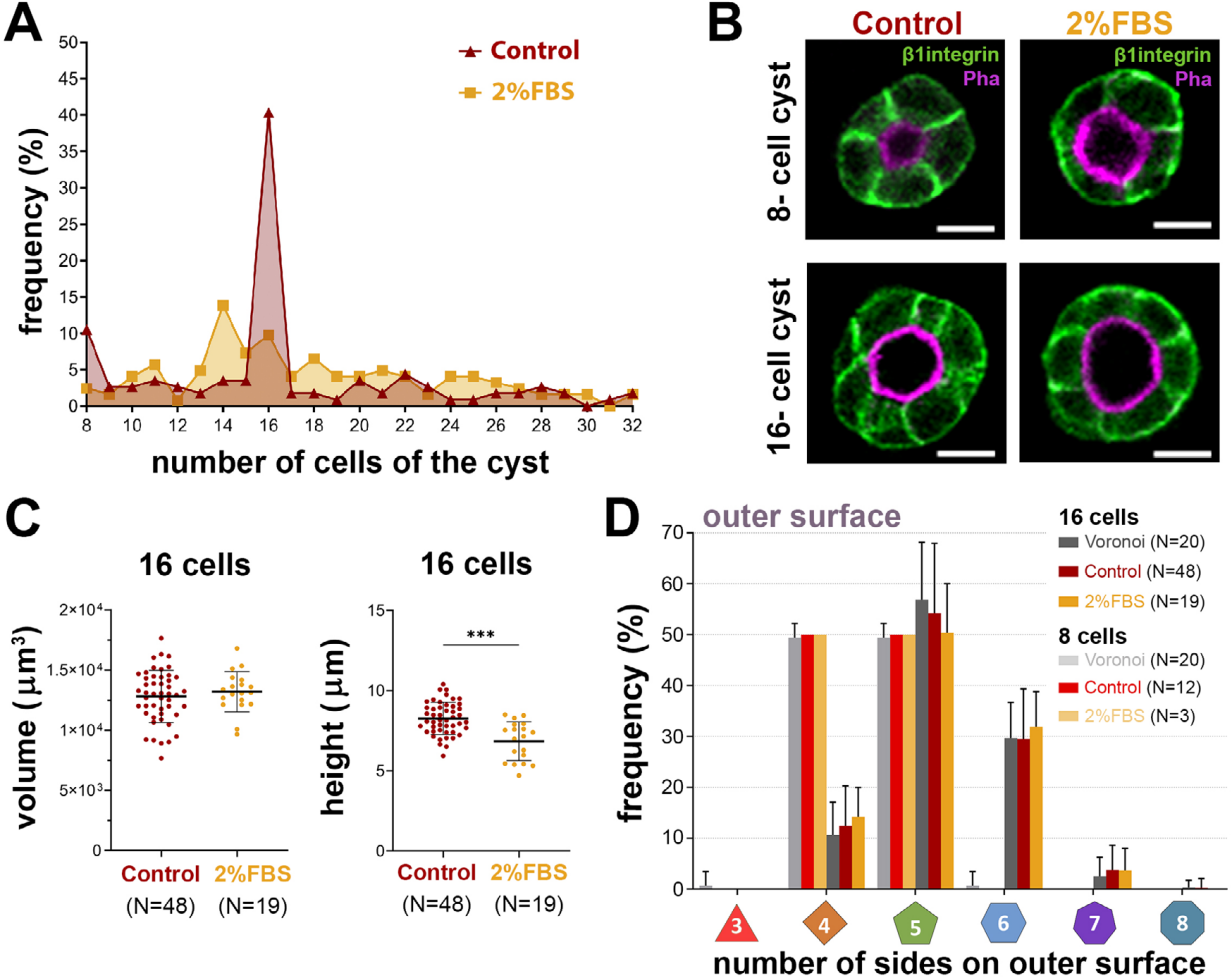
Tissue organisation in cyst with high curvature per cell‐patch. (A) Frequency of cysts formed by different cell numbers in the range from 8 to 32 cells (Control and 2%FBS cysts grown during 3 and 4 days respectively). (B) Representative confocal sections of the equatorial plane of 8‐cell Control and 2%FBS cysts (top) and 16‐cell Control and 2%FBS cysts (bottom). The phalloidin staining delineates the outline of the lumen (magenta), while β1‐integrin the basolateral cell domain (green). Scale bar, 10 µm. (C) 16‐cell 2%FBS cysts show no significant difference in volume (left) compared with Control cysts, but exhibit a significant decrease in cell height (right). Each dot represents a single cyst. Bars indicate the mean ± s.d. ***p < 0.001 by two‐tailed Student's *t*‐test. (D) Oblate spheroid Voronoi models, Control cysts, and 2%FBS cysts present similar average polygon distributions in both 8‐cell (lighter colours) and 16‐cell comparisons. Triangles are indicated in red for all figures. Data are shown as mean + s.d. There are no significant differences in the 16‐cell group by PERMANOVA. The statistical comparison was omitted for 8 cells (zero variance).

### 2.3 Polygon Configurations Reflect Shell Organisation

The inherent variance in polygon frequencies observed in small cysts, even among those with the same number of cells, led us to explore alternative methods for characterising shell organisation. In quasi‐regular polyhedral shells, polygon centres are evenly spaced, leading to specific, predictable configurations; for example, 12 pentagons in a dodecahedron or 12 pentagons with 20 hexagons in a Buckminsterfullerene (Figure 1F). These polygon configurations are consistent with the Euler's condition that applies to polyhedra where each vertex is formed by the intersection of three edges: the total number of edges, *E*, must satisfy the condition *E = 3n − 6*, where *n* is the number of polygons [40, 52]. As a consequence, only a restricted set of polygon configurations can arise in the cysts. For the 8‐cell case we identified 40 *theoretical configurations* compatible with Euler's rule to shape a polyhedron (Table S3; Methods). However, as shown in Figure 4D, all the cysts analysed presented a polygon configuration with four quadrilaterals and four pentagons (represented as 0‐4‐4‐0‐0) (Figure 5A). This configuration was also the most frequent in the oblate spheroid Voronoi model (92 out of 100 simulations; Figure 5B,C; see also Table S3). For the 16‐cell case, we identified 619 *theoretical configurations* compatible with Euler's rule to shape a polyhedron (Table S3; Methods). We analysed 48 Control cysts and found that just 8 polygon configurations accounted for 87.3% of all samples (Figure 5C; see also Table S3). The same configurations were also the most common in 100 simulations using the 3D Voronoi model (Figure 5B,C). These frequent configurations were enriched in pentagons while triangles and octagons were absent. The divergences in polygon types observed in the configuration analysis provide an explanation for the high variance in polygon frequencies found in small cysts (Figures 2E and 3D, see also Table S2).

**FIGURE 5:**
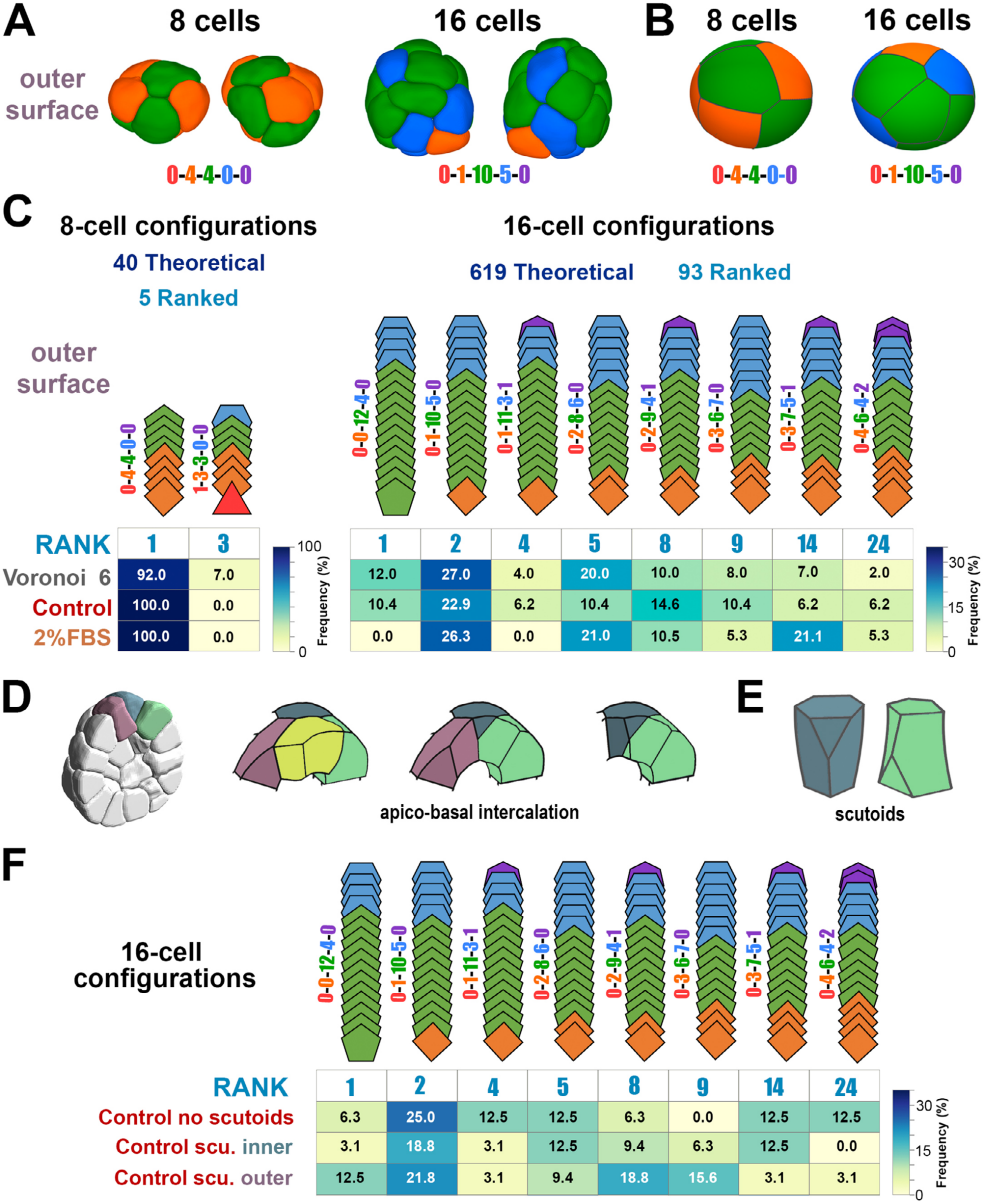
Polygon configurations in small biological shells. (A) 3D reconstructions (front and back views) of 8‐cell (left) and 16‐cell (right) cysts colour‐coded by the number of cell neighbours. Adjacent numbers indicate the count of each polygon type, following the colour scheme. (B) Representative 8‐cell (left) and 16‐cell (right) 3D oblate spheroid Voronoi models. (C) Top: Colour‐coded outer polygonal configurations for the 8‐cell and 16‐cell cases on the outer cyst surface. For the 8‐cell case, 5 of the 40 *theoretical configurations* were identified among 100,000 analytical Voronoi realisations (Methods). For the 16‐cell case, 93 of the 619 *theoretical configurations* appeared in the analytical realisations, with eight of those configurations representing 87.3% of the observed cases for MDCK Control cysts. In both cases, configurations are ordered from left to right according to their ranking by the compactness descriptor. The configurations shown are those present with a frequency higher than 6% in the outer surface of the Control cysts. The same set of configurations is shown in Figure 5F and Figure S8A. Bottom: For each configuration, the numerical rank position is indicated above a heatmap showing the frequency with which each configuration occurs in the 3D Voronoi model (Voronoi 6), in Control cysts, and in 2%FBS cysts. (D) At the left, it is shown a 3D reconstruction of a MDCK cyst showing 4 cells participating in an apico‐basal intercalation. On the right, a schematic of these four cells showing their organisation and contacts along the apico‐basal axis. (E) Drawing of two idealised scutoids. (F) Analogous to (C), the heatmap displays the ten most frequent configurations observed across inner and outer surfaces, collectively accounting for over 80% of all cysts. Frequencies are shown for (from top to bottom): cysts without scutoids, where the inner and outer surfaces have identical configurations (Control no scutoids); the inner surface of cysts with scutoids (Control scu. inner); and the outer surface of cysts with scutoids (Control scu. outer).

A key question arising from these results is why, in the case of 16‐cell samples, only a small subset of the 619 *theoretical configurations* appears frequently on the real cysts. Epithelial cyst surfaces are largely composed of cells with relatively homogeneous areas (low standard deviation in cell area) and quasi‐regular polygonal shapes (low variation in side lengths) (Figure 2E and Figure S2C). Since regular polygons maximise their area‐to‐perimeter ratio [53], we hypothesised that the observed arrangements would correspond to those with higher average values of a dimensionless *compactness descriptor* ($\sqrt{area}/perimeter$). This descriptor is the inverse of the ‘shape index’ ($perimeter/\sqrt{area}$), widely used in tissue mechanics [54]. However, the *compactness descriptor* formulation was adopted so that higher numerical values directly and intuitively scale with increasing geometric compactness, aligning with classical shape‐characterisation metrics [55].

First, we aimed to identify which of the *theoretical configurations* actually occurred in epithelial shells organised similarly to cysts. To achieve this, we designed a method that efficiently generated 100 000 realisations of spherical surfaces reproducing cyst‐like organisation (Voronoi 6 topology, Methods). This method also enabled the calculation of a *compactness descriptor* for each resulting polygon configuration. This allowed us to rank all unique configurations by compactness. Remarkably, only 5 unique configurations appeared among the 40 theoretical 8‐cell arrangements, and 93 among the 619 theoretical 16‐cell ones (Table S3). These subsets are referred to as the *ranked configurations*. The five most frequent configurations observed in 16‐cell cysts fell within the top 10 positions, and the unique configuration observed in 8‐cell cysts was the top one (Figure 5C; see also Table S3). Our 3D oblate spheroid Voronoi model (Voronoi 6) and Control cysts presented similar proportions of each configuration (Figure 5C). We also observed that cell configurations remained consistent across different geometries of the 3D Voronoi model (Figure S7A). In addition, high‐ranked configurations presented a large similarity in the polygon‐polygon contact matrices (Figure S7B–G, see Methods).

We tested if the limited range of configurations observed were also extended to 2%FBS cysts. As expected, for this condition, the three 8‐cell cysts analysed adopted the top‐ranked configuration. The 16‐cell samples (N = 19) displayed eight distinct configurations, seven of which coincided with those observed in the Control group, confirming the same predominant presence of high *ranked configurations* (Figure 5C; see also Table S3). These findings support our initial hypothesis that the configurations on the outer surfaces of small cysts maximise the *compactness descriptor*, regardless of the modification of relevant biological conditions.

### 2.4 Scutoids Enable Efficient Reorganisation on the Outer Surface of Epithelial Cysts

Cells within MDCK cysts frequently adopt a scutoidal geometry, facilitating apico‐basal intercalations [29]. These intercalations alter cellular connectivity, thereby allowing the inner and outer surfaces of the tissue to exhibit distinct polygonal distributions (Figure 5D,E). We examined the influence of scutoids on polygonal configurations in 16‐cell Control cysts, which were classified as containing (32 out of 48) or lacking scutoids (16 out of 48). The common configurations found in the outer surface of cysts containing scutoids were slightly different to those observed in cysts without presence of scutoids (Figure 5F). Next, we compared the inner and outer surfaces in relation to scutoid presence, noting that in cysts with scutoids, apical and basal surfaces must differ due to cell neighbour exchanges along the apico‐basal axis. The inner surface of cysts with scutoids showed a lower proportion of the frequent configurations and included some arrangements that ranked lower according to the *compactness descriptor* (Figure 5F; see also Table S3). Furthermore, categorising these events revealed that cysts with multiple apico‐basal intercalations exhibited a higher prevalence of low‐ranked polygonal configurations on the inner surface and high‐ranked configurations on the outer surface than those with a single transition (Figure S8B). At the single‐cell level, tracking of apico‐basal intercalations demonstrated that scutoidal cells undergo targeted structural remodelling given rise to an outer surface (basal) dominated by pentagons and hexagons (84.3% of scutoidal cells) (Figure S8C). This reorganisation features a marked enrichment in outer surface pentagons (from 33.2% in the inner surface to 53.5% in the outer surface), while the hexagons population remains largely conserved. This correlates with the finding that, at the outer surface, scutoid‐containing cysts adopt higher‐ranked configurations, which are characterised by a greater proportion of pentagons (Figure S8C). We also analysed the 3D cell packing in cysts cultured with 2%FBS and found a higher proportion of cysts without scutoids (13 of 19 samples). In 2%FBS cysts that exhibited scutoids, configurations in the outer surface shifted toward top‐ranked configurations, similar to that observed in the equivalent case for Control cysts (Figure 5F and Figure S8A). These findings suggest that while the inner surface can contain unusual, low‐ranked configurations, they are resolved into top‐ranked on the outer surface (see Discussion).

### 2.5 Organisational Constraints in Early Mouse Embryos are Similar to Those Observed in Cysts

Most epithelial cells share fundamental properties such as adhesion, turgidity and the minimisation of surface tension, allowing for comparison with the mathematical concept represented by the Voronoi diagrams [6, 8]. Therefore, we tested the applicability of our model to simulate other types of epithelial shells, such as the mouse embryos at 8‐ and 16‐cell stages (Figure 6A and Figure S9). These stages involve tightly coordinated developmental events essential for early tissue organisation. The first major morphogenetic process is compaction, which begins at the 8‐cell stage and leads to stronger cell–cell adhesion, the establishment of apico‐basal polarity, and overall smoothing of the embryo surface [56, 57]. During the transition from 8 to 16 cells, cell divisions occur asynchronously and are coupled with dynamic cell rearrangements [44, 58]. These rearrangements are essential for maintaining tissue integrity and enabling the first lineage segregation events. During this process, cells differentiate into either the inner cell mass (a pluripotent cluster of cells that will form the complete embryo) or the trophectoderm progenitors (the outer layer that gives rise to the extraembryonic tissues). Therefore, the analysis of cellular topology at the embryo surface can provide insights into the principles underlying early tissue organisation. To analyse this process in a comprehensive manner, we designed a specific Voronoi model to capture the absence of a lumen in the early stages of mammalian embryogenesis (Figure 6B; Methods). To this end, we located Voronoi seeds within the interior of the solid sphere, generating a clipped 3D Euclidean Voronoi model (Figure 6B; Methods). Given the broad distribution of cell areas observed in these embryos and based on previous studies linking polygonal and area distributions in epithelial tissues [6], we tested various conditions to best reproduce the relative proportions of cells in the embryo's interior and surface (Figure 6C and Figure S9A; Methods; see also Table S4). Specifically, we initialised the model with randomly distributed seeds inside the spheroid and applied Lloyd's algorithm to iteratively homogenise inter‐seed distances, enabling us to analyse resulting cell distributions. The most accurate match for outer organisation and total number of neighbours occurred when seeds were heterogeneously distributed (Voronoi 2, 100 realisations) compared to a more homogeneous arrangement of the seeds (Voronoi 6, 100 realisations) (Figure 6C,D, and Figure S9C,D; Methods). We then compared outer‐surface configurations between mouse embryos and Voronoi simulations (Figure 6E–H). The 8‐cell Voronoi model captured the real sample configurations in 20 out of 23 cases (Table S5). Embryos with fifteen outer cells (here, defined as those in contact with the embryo surface) and one inner cell (completely enclosed) displayed surfaces composed of polygons of different sizes and similar side lengths, with 6 out of 7 configurations matching those observed in the corresponding Voronoi model (Figure 6E,G; see also Table S5). These configurations also ranked highly according to the *compactness descriptor* (Figure 6G). In contrast, embryos with 16 outer cells presented a mix of configurations: 5 out 12 shared with Voronoi simulations (Figure 6E,H; see also Table S5), and the rest absent from the 100 simulated cases. Examination of these discrepancies revealed the presence of cells in intermediate states between outer and inner positions, as well as overlapping cells (Figure 6F). Other cases with different arrangements may not even satisfy Euler's condition, being out of the *ranked configurations* (Table S5). These rare cases involve images whose segmentation indicates the presence of a cell that touches the surface in two separated places, having different cell contacts for each surface patch (Figure S9B). Our findings suggest that the constraints derived from the closed‐shell condition are the main driver of cell contact patterns. For this reason, the 3D Voronoi models can predict their organisation even considering different structures (such as cysts and embryos) and number of elements (cells).

**FIGURE 6:**
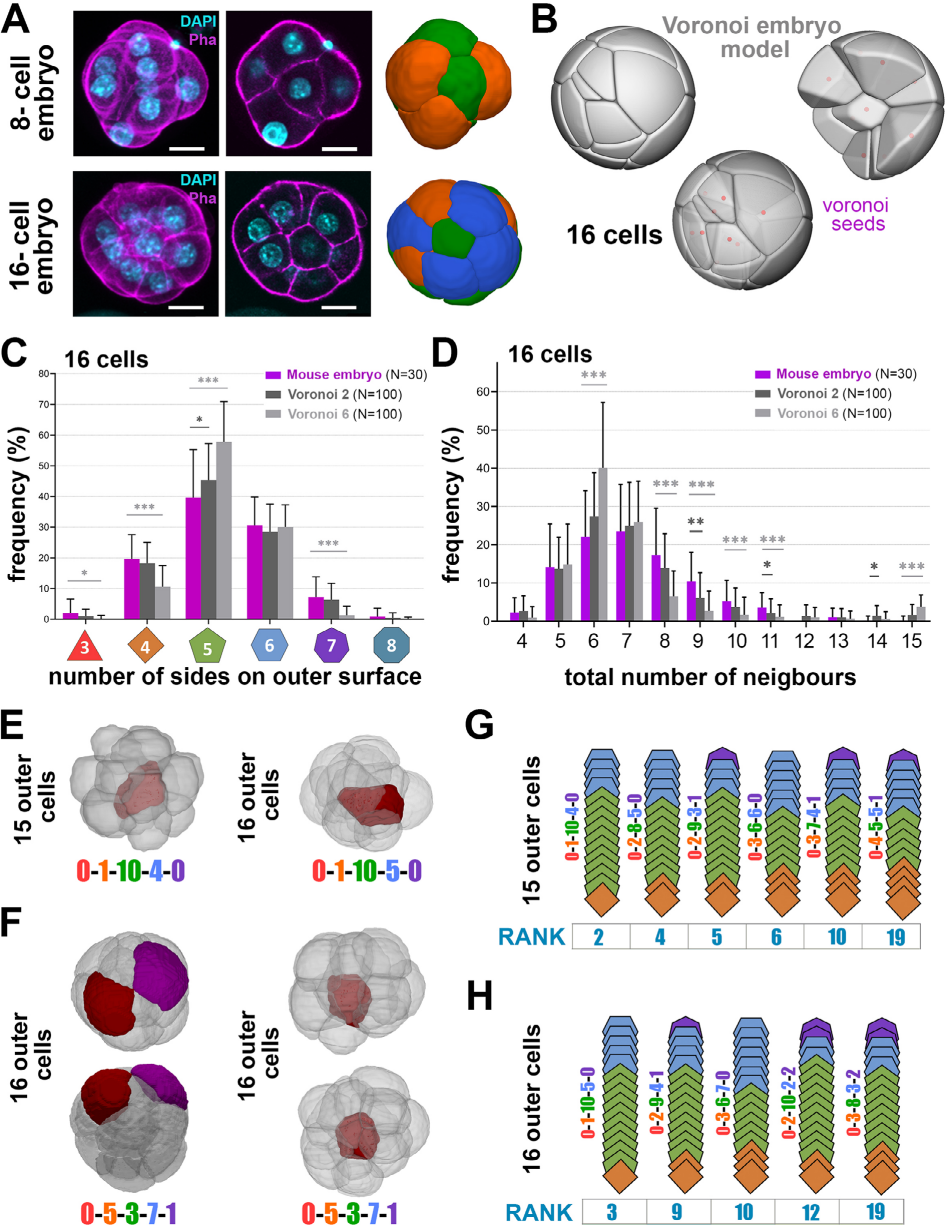
Organisational constraints in early mouse embryos. (A) From left to right, maximum Z‐projection (DAPI, cyan; phalloidin, magenta), equatorial section, and 3D reconstruction of an 8‐cell embryo (top) and a 16‐cell embryo (bottom). Scale bar, 20 µm. (B) 3D representation of a Voronoi embryo simulation. Left: full simulation. Centre: front cells shown semi‐transparent to reveal Voronoi seeds (pink). Right: some front cells have been removed to expose the inner organisation. (C,D) Comparison of 16‐cell embryos (magenta) and 16‐cell Voronoi model with different Lloyd iterations (Voronoi 2: 1 iteration, dark grey; Voronoi 6: 5 iterations, light grey). Data show the average polygon distribution on the outer surface (C) and the average total number of neighbours (D) with error bars indicating the s.d. Statistical significance of the overall distributions was evaluated by PERMANOVA (*p* < 0.001, for comparison of mouse embryos with Voronoi 6) followed by post‐hoc Kruskal–Wallis tests with Dunn's multiple comparisons correction to identify differences between polygon classes. *p < 0.05; **p < 0.01; ***p < 0.001. (E) Left: 3D representation of a 16‐cell embryo with 15 cells on the outer surface (inner cell, dark red). Right: 3D representation of a 16‐cell embryo with all the cells in contact with the outer surface. The central part of the embryo is occupied by cells that touch the outer surface like the one highlighted in dark red. Below both 3D representations, configuration is shown with the polygon colour code. (F) Left: Two vertically arranged views of the same 3D representation of a 16‐cell embryo. Highlighted cells contact and partially overlap the underlying cells. Right: Two vertically arranged views of the same 3D representation of a 16‐cell embryo, showing a blastomere transitioning to an inner position while keeping minimal contact with the embryo surface. These events enable configurations not observed in the models (100 simulations), which is consistent with the low frequency at which this configuration occurs in the ranking (approximately ∼1% of the 100 000 realisations). Below each 3D representation, the configuration is shown with the polygon colour‐code. (G,H) Set of configurations identified in embryos with 15 outer cells (G) and 16 outer cells (H), ordered from left to right by compactness ranking (see Methods). Top‐ranked configurations are shown, collectively accounting for over 80% of the samples.

## 3 Discussion

Epithelial tissues are characterised by basic properties of the component cells such as adhesion, turgidity, and surface tension minimisation. Cells tend to adopt regular polygonal shapes that maximise the area‐to‐perimeter ratio and minimise the energetic cost of the system, as classically formalised in vertex models [9, 59]. As a consequence, the tissue forms a tessellation that imposes geometric and topological constraints limiting its possible organisation. Here, we have dissected how the closed‐shell condition of cysts and mouse embryos introduces additional constraints that further determine their polygon distribution. While planar surfaces can be perfectly tiled with hexagons, this is not possible in a closed shell, where positive curvature must be introduced. In this context, pentagons represent the minimal deviation from hexagonal tilling needed to close the surface. Derived from Euler's theorem, fully covering a sphere with only hexagons and pentagons requires exactly twelve pentagons, as observed in soccer balls and fullerene molecules [36, 37, 40, 60]. Using our 3D Voronoi model, we show that high cell patch curvature, associated with a low number of cells tessellating the surface, leads to an increased proportion of pentagons [45, 47]. Consistently, we find that this principle holds in both cultured cysts and mouse embryos.

The clear relationship between the number of cells and the curvature restrictions has led to an in‐depth analysis of numerous samples with a small number of cells, focusing on the polygonal configurations permitted by Euler's condition. Gauss‐Bonnet and Euler theorems impose constraints on the epithelial organisation. These constraints include the curvature–cell number relationship, reflected by the higher proportion of pentagonal cells in small cysts, as well as the limited number of topologically feasible configurations, which decreases as cyst size becomes smaller. However, while these theorems define the space of possible topological states, they do not predict the probability of individual polygonal configurations. In that sense, a main result of our study is the Voronoi‐based enumeration of the most likely configurations within this theoretical space and their subsequent ranking according to compactness. We cannot definitively state that the remaining *theoretical configurations* are geometrically impossible, but they are extremely improbable under the constraints of the analytical Voronoi model (and the biological observations) where there is a high homogeneity of cell surface areas (Figures S1B and S2C). While we state that the compactness ranking is primarily a geometric descriptor, given the presence of relatively homogeneous cell areas, higher compactness values correspond to shorter cell perimeters. In epithelial vertex models, shorter perimeters are associated with lower perimeter‐related energetic costs [6, 8]; therefore, configurations with higher compactness may correspond to energetically more favourable states under this framework.

In 8‐ and 16‐cell cysts, we found that only a small subset of the theoretically possible configurations was observed. Despite some variations in frequencies, Control, 2%FBS, and Voronoi shells exhibited a remarkably similar set of configurations (Figure 5; see also Table S3). Notably, the configurations found in 8‐ and 16‐cell cysts ranked highly according to the *compactness descriptor*. At the 8‐cell stage, all Control cysts adopted the top‐ranked configuration of four quadrilaterals and four pentagons, highlighting the strong topological limitations. Interestingly, although high‐compactness configurations predominated, some top‐ranked arrangements were absent in both cysts and the corresponding Voronoi models. In 16‐cell shells, this is the case of some configurations including two heptagons (ranks 3, 6, 17), triangles (ranks 7, 10, 12, 17, 18), or even octagons (rank 12) (Table S3). We propose that the high degree of cell area homogeneity in the outer surface of these small cysts (and in their corresponding Voronoi models) prevents the formation of configurations that require pronounced asymmetries in cell size [61]. Together, our results suggest that the interplay between cell area uniformity and compactness may act as an important determinant of the limited topological diversity observed in small epithelial shells.

Our results indicate that, particularly in small cysts, the frequency of configurations offers a more accurate and comprehensive representation of topological organisation than classical average neighbour distributions, even capturing aspects of the 3D organisation. We observed a larger frequency of higher‐ranked configurations on the outer surface of both Control and 2%FBS cysts with scutoids (Figure 5F and Figure S8A). A possible explanation for the higher variability (and lower rankings) on the inner surface is that lumen morphology and formation are influenced by mechanical factors, such as variations in hydrostatic pressure and cortical forces [62, 63]. Our data suggests that, in closed 3D systems, scutoids may function as a structural adaptor that facilitates the formation of optimal high‐ranked configurations on the outer surface. While these findings are currently descriptive and the precise mechanism driving the differences between the inner and outer surfaces remains unknown, the fact that scutoids enable the enrichment of pentagons on the outer surface suggests this potential link (Figure S8B,C).

In early mouse embryos, non‐luminal compact epithelial shells, our Voronoi model can also reproduce the configurations that appear on the outer surface. At the 8‐cell stage the configurations 0‐4‐4‐0‐0 and 1‐3‐3‐1‐0 represent more than 73% of the arrangements in both embryos and the Voronoi embryo model. These results are compatible with previous findings showing that 8‐cell embryos tend to adopt a limited set of preferred rigid 3D packings that favour robust and successful morphogenesis [44]. By contrast, 16‐cell embryos exhibit substantially greater variability compared with cysts, with no single configuration dominating. The additional degree of spatial freedom, given by the absence of a lumen, allows a broader spectrum of arrangements, from highly ordered to more irregular distributions. In the case of the 16‐cell embryos with 15 cells contacting the surface, the inner cell excludes the outer cells from the centre of the shell, promoting a more regular outer configuration as lumen does in cysts. This explanation is consistent with recent findings linking nuclear rigidity and intercellular spacing to the regulation of cell packing arrangements [47]. We propose that the uniform cell‐spacing, and thereby the homogeneous area distribution, prevents “theoretical” configurations with a high number of triangles or octagons. In contrast, embryos with 16 cells on the outer surface displayed greater organisational heterogeneity. This reflects the highly dynamic nature of the 16‐cell stage embryo, where cells divide and sort at variable angles, which determines their eventual positioning as outer, intermediate, or inner cells [58, 64]. The 3D Voronoi model accurately predicts the configurations from embryos with 8 or 15 outer‐surface cells. However, the accuracy is reduced in some embryos with 16 cells in the outer surface, where intermediate cells can cause ambiguous configurations (Figure 6F). Taking all our results together, we observe that predictive power of the model is limited in tissues undergoing active 3D reorganisation (Figure 6F and Figures S8D and S9B and Table S5).

Our 3D Voronoi model predicts the organisation of epithelial cysts, even when cultured under altered biological conditions, such as reduced FBS concentration or disruption of actomyosin contractility. These results suggest that, in the epithelial shells, the curvature experienced by each cell is a major contributor to surface organisation, with an influence that appears to surpass the biological perturbations. Together, these results indicate that the geometry, topology and curvature of epithelial shells confer a high degree of robustness to their cellular organisation, a property that is likely to be biologically relevant given the central role of these structures in morphogenesis. Indeed, closed shells often serve as intermediate structures at early stages during development, where stable tissue organisation is required to support subsequent morphogenetic events [65]. Since epithelial organisation directly influences cell–cell connectivity and juxtacrine signalling [66, 67], this robustness ensures that such processes are not substantially perturbed. The resulting preservation of characteristic polygonal configurations, including the presence of clusters of pentagons in small shells, could also be relevant for morphogenesis, as local enrichments of non‐hexagonal polygons have been associated with mechanical instabilities and morphological transitions in epithelia [68]. In summary, by framing curvature, geometric and topological constraints in a general theoretical context, our work provides a foundation for investigating early tissue pattern formation across animal species and shed light on how 3D organisation is regulated during the initial stages of embryogenesis.

## 4 Methods

### 4.1 MDCK Cyst Cell Culture

MDCK‐II (*Madin‐Darby canine kidney*) cells were maintained in minimum essential medium (MEM) containing GlutaMAX (Gibco) and supplemented with 10% fetal bovine serum (FBS), 100 U/mL penicillin and 100 µg/ml streptomycin, in a 5% CO_2_ humidified incubator at 37°C. For cyst formation, MDCK cells (2500 cells/well) were suspended in complete medium containing 2% Matrigel (Corning, Life Sciences). Cell suspension was plated in a four‐well culture slide (Corning, Life Sciences) on a thin layer coating of 100% Matrigel. The plates were kept at 37°C in a humidified atmosphere of 5% CO_2_ for 3, 4, 7, or 10 days and the medium was changed every 2 days. To study the effect of the proliferation and cell tension on the distribution of polygonal cell types, MDCK cysts were cultured respectively in presence of low FBS concentration (2%FBS) and of 20 µM ROCK Inhibitor (Y27632, Wako) (10 mm stock in sterile ultra‐pure water). In the case of low‐serum experiments, cysts were treated from the seeding to days 4, 7, and 10. For the ROCK inhibition, cysts were treated with Y27632 from the seeding to 3 or 4 days. During the treatments the medium was changed every 2 days.

For growth curve analysis under control (10% FBS) and low nutrient conditions (2%FBS), 4000 MDCK‐II cells were seeded into 24‐well culture plates (Thermo Scientific, Nunclon Delta Surface). Three replicates were cultured for each time point to ensure uniform initial cell density. Cells were trypsinised every 24 h and counted using the vital staining Trypan blue (Sigma–Aldrich) for 10 days. Cell counting was performed by LUNA Automated Cell Counter (Logos Biosystems).

### 4.2 MDCK Cyst Immunostaining and Confocal Imaging

Cysts grown on four‐well chamber slides (Corning, Life Science) were fixed with 4% paraformaldehyde in PBS and permeabilised with 0.5% TritonX‐100 in Dulbecco's Phosphate Buffered Saline (DPBS, Sigma‐Aldrich) for 15 min at room temperature (RT). After blocking with a solution of 0.02% Saponin (Sigma) and 3% BSA (Applichem) in DPBS for 2 h at RT, cysts were incubated overnight at 4°C with anti‐β‐catenin antibody (rabbit, Sigma–Aldrich) and anti‐β1‐integrin (rat, Developmental Studies Hybridoma Bank, DSHB) both diluted 1:1000 in DPBS‐0.02% Saponin‐3% BSA (staining solution). To demonstrate the effect of ROCK activity inhibition, we used the anti‐Phospho Myosin Light Chain 2 (pMLC Ser19) primary antibody (Rb, Cell Signaling). The following day, the cysts were washed with staining solution (3x, 5 min each) and incubated for 90 min at RT in this solution plus anti‐rabbit and anti‐rat secondary antibodies conjugated to Alexa Fluor (1:800, Molecular Probes) and phalloidin‐Alexa‐Fluor 647 (0.08 µm, Invitrogen). After washing with DPBS, plastic chambers were removed from microscope slides and coverslips were mounted onto slides using Fluoromount‐G mounting solution (SouthernBiotech).

Most of the cysts were imaged using a Nikon Eclipse Ti‐E laser scanning confocal microscope. Images were captured (from top to bottom) using 20x oil objective (0.75 NA), with a Z‐step of 0.7 µm per slice, scan speed of 0.5 ms and exported as 1024 × 1024 pixels *.nd2* files with an XY resolution of 1.6248 pixels per micron. Some images of 3‐day control cysts and all the samples from the ROCK inhibitor experiments were acquired using a Leica Stellaris 8 laser scanning confocal microscope equipped with a 20× glycerol objective (0.75 NA) and applying the same imaging settings described above. These images were exported as 1024 × 1024 pixel *.lif* files.

### 4.3 Mouse Embryo Culture and Imaging

Mouse embryo culture and imaging were performed as previously described [64].

#### 4.3.1 Fixed Embryos

Embryos obtained by mating CD1 wild‐type mice, were harvested at the desired developmental stage, and fixed in 4% paraformaldehyde in PBS for 10–15 min at room temperature. Samples were then permeabilised in 0.5% Triton X‐100 in PBS for 20–30 min, followed by blocking in 10% fetal bovine serum (FBS), 0.1% Triton X‐100 in PBS for 1 h. For immunostaining, embryos were incubated overnight at 4°C with primary antibodies diluted in blocking solution. The following morning, embryos were washed in PBS and incubated with appropriate secondary antibodies for 1–2 h at room temperature. For F‐actin staining, embryos were incubated in Alexa Fluor 647 Phalloidin (1:500, Invitrogen, A12380) and DAPI (1:1000) diluted in blocking solution for 30 min to 1 h at room temperature, in place of the primary antibody incubation step. After staining, embryos were imaged using a Leica SP5 II confocal microscope, acquiring Z‐stacks at 1–2 µm intervals per wavelength.

#### 4.3.2 Live Imaging

Membrane‐GFP transgenic mice were produced by crossing the R26‐mTmG line (JAX #007576) with a Nestin‐cre mouse to permanently remove the floxed membrane‐localised tdTomato in the germline. Embryos obtained mating ubiquitously expressing membrane‐GFP transgenic male mice with CD1 wild‐type female mice, were harvested and cultured as previously described [64]. Cultured embryos were imaged on an inverted Leica SP5 II MP system with a Chameleon Ti:Sapphire laser (Coherent Inc.) enclosed by a Ludin Life Imaging chamber incubation system maintained at 37°C and 5% CO2. Z‐stacks were collected every 7–10 min with 2 µm sections (∼55 total) at 890 nm for GFP. Movies were reconstructed using IMARIS software (Bitplane, AG). For the analysis of polygonal distributions and tissue configurations, specific time points were selected from time‐lapse movies corresponding to the 8‐ and 16‐cell stages. For the 8‐cell stage, frames were chosen when embryos exhibited clear compaction. For the 16‐cell stage, frames were selected when the tissue appeared stabilised and no cell divisions were occurring.

### 4.4 Post‐Acquisition Processing for Figure Images

For MDCK cyst and embryo images, contrast/brightness adjustments were performed using Fiji (ImageJ). The ND‐SAFIR tool was applied for denoising only in Figure 1E.

### 4.5 Image Segmentation and Feature Extraction

We acquired between 10–30 images from each experimental setup: condition and time of cyst culture. In these images we processed all the single‐cell, single‐lumen cysts as described in ref. [29]. Confocal image stacks of MDCK cysts and mouse embryos were segmented at the single‐cell level through the following pipeline:

1. Preprocessing: Image contrast was standardised using custom Fiji macros. Contrast enhancement was applied via the ‘Enhance Contrast’ function with a 0.3% saturation threshold to limit pixel saturation, followed by conversion to 8‐bit to simplify downstream segmentation.
2. Segmentation: Cells were segmented using CartoCell, a pretrained instance segmentation model specialised for epithelial tissues [29]. The model employs a custom 3D residual U‐Net (3D ResU‐Net) architecture and was trained using the BiaPy platform [69]. Model inference produces a three‐channel output (cell membranes, cell bodies, and tissue volume).
3. Postprocessing: The Watershed algorithm separates individual cells, while a Voronoi‐based approach fills intercellular gaps based on proximity, enabling accurate cell instance segmentation. Although originally trained on cyst confocal images, the model performs reliably on embryo datasets.
4. Proofreading: Instance segmentations were manually curated by experts to ensure accurate cell morphology and correct cell–cell connectivity. In the case of mouse embryos, polar bodies were manually removed from the segmentation.
5. Feature Extraction: A range of geometric and topological parameters was quantified using a custom MATLAB library adapted from the CartoCell toolkit [29], which we originally developed to extract features at the cell level from epithelial cysts. The code was adapted for application to embryos, which lack a central lumen, enabling accurate feature quantification in these tissues. For both tissue types, cysts and embryos, we consistently quantified cell perimeter, surface area, volume, and the total number of cell neighbours. Cell neighbours were calculated both on the outer cell surface and given their total 3D cell contacts. In cysts, additional measurements were obtained from the inner surface, including cell area, perimeter, and number of neighbours. To assess apico‐basal intercalations, we identified cells that exchanged neighbours along the apico‐basal axis, a process indicative of scutoid formation.

### 4.6 Computer Simulations

To identify topologically feasible configurations capable of forming polyhedral shells, we first employed an in‐house Python algorithm to systematically enumerate all possible polyhedral configurations with 8 or 16 faces. The algorithm evaluates combinations of polygonal faces ranging from triangles to octagons and retains only those that satisfy Euler's theorem. According to this theorem, for a closed surface tiled by polygonal cells where each vertex is formed by the intersection of three edges, the total number of edges, *E*, must satisfy *E = 3n‐6*, where *n* is the number of cells. Using this approach, we identified 40 valid configurations for 8‐cell shells and 619 for 16‐cell shells (Table S3), hereafter termed *theoretical configurations*.

To investigate the geometric realisation and relative occurrence of these theoretically feasible configurations, we developed three variations of Voronoi models to simulate biological shells: (1) shells with a lumen, resembling epithelial cysts; (2) solid shells, analogous to mouse embryos; and (3) an analytical version to study of the possible polygonal configurations on spherical surfaces. A Voronoi diagram partitions space into regions around each generator seed point, so that all points within a region are closer to that seed than to any other. These models were designed to approximate the organisation of epithelial shells, providing a theoretical baseline for comparison with biological tissues. The models were implemented in MATLAB using a combination of custom code and modified third‐party algorithms. The Voronoi models used here extend previous implementations developed by our group [11, 23] adapting it to the analyses of cysts and mouse embryos.

#### 4.6.1 Geodesic 3D Voronoi on Spheroidal Surfaces

We modelled the structural and organisational properties of MDCK cysts using geodesic 3D Voronoi tessellations on spheroidal surfaces. The epithelial cysts analysed in this study are characterised by a monolayer epithelium forming a shell, with a basal surface facing the exterior and an inner (apical) surface enclosing a lumen (Figure 1). These shells exhibit diverse morphologies with varying frequencies, including spheres (4.0%), oblate spheroids (34.3%), prolate spheroids (18.1%), ellipsoids (39.9%). Finally, we categorised a small fraction of cysts as exhibiting negative curvature (3.7%) [29]. To parameterise the spheroidal model, representative axis lengths for the inner surface were derived from actual lumen measurements. Specifically, we calculated the principal axis lengths of cyst lumina belonging to the top 25% of cases (N = 117) with the highest convex hull‐to‐volume ratios. This criterion was chosen to minimise the influence of local inhomogeneities and irregularities that could influence the principal axes length calculation. The analysis yielded a median minor‐to‐major axis ratio of 0.75 (mean: 0.72 ± 0.03), which was adopted for the simulations.

We performed 20 simulations per cell number on both spherical and oblate spheroidal geometries to generate in silico epithelial cysts, with the exception of 8 and 16 cells where 100 simulations were conducted across all three spherical, oblate, and prolate geometries for a more detailed study of the inner and outer surfaces (Table S1).

Each surface was shaped by a mesh of equilateral triangles at a resolution of 10^6^ triangles. For each simulation, *N* seed points (representing *N* cells) were randomly distributed on the vertices of the inner surface mesh. Seeds were placed one by one while maintaining a Poisson‐disk sampling rule, requiring a minimum distance of 50 vertices from all previously placed seeds. If no valid position met this condition, the minimum spacing was gradually reduced until a suitable location was found.

Then, we generated the Voronoi tessellation based on geodesic distances, using the Fast Marching method described by [70]. This technique facilitated relatively efficient tessellation on curved surfaces, which is particularly advantageous when *N* is large.

Subsequently, tessellations were iteratively relaxed using Lloyd's algorithm (up to five iterations for Voronoi 6), where each seed was replaced by the centroid of its corresponding Voronoi cell. This approach was designed to imitate the conserved organisation of epithelial cells previously described in planar surfaces [3, 6, 46]. In the terminology used, Voronoi X corresponds to X‐1 Lloyd iterations.

After defining the inner surface, the outer surface was generated by constructing a concentric sphere or spheroid with corresponding geometric properties at a specified distance given by the cell height. Based on CartoCell data [29] ranging from 8 to 450‐cell tissues, a cell number–dependent outer‐to‐inner radius ratio was established using the regression (R^2^ = 0.89):

$$\mathrm{Outer}-\mathrm{to}-\mathrm{inner}\;\;\mathrm{radius}\;\mathrm{ratio}=2.978\cdot N^{\left(-0.153\right)}$$

where *N* is the number of cells. Seeds from the inner surface were then projected outward along the local normal vectors until they intersected the outer surface, where the outer Voronoi tessellation was constructed.

From this model, we obtained the geodesic distance from each mesh point to each seed, the edges that define the Voronoi tessellation, and the set of triangles associated with each Voronoi cell patch. This information enabled the measurement of each cell's surface area and perimeter, as well as the identification and quantification of neighbouring cells. These data enabled the subsequent classification of cells according to their polygonal shape.

#### 4.6.2 Euclidean 3D Voronoi on Spherical Shells

To model 8‐ and 16‐cell mouse embryos, which lack a lumen and are approximately spherical, we adapted our previous approach using Euclidean 3D Voronoi tessellations on spherical shells. First, a voxel‐wise 3D mask of a sphere with radius *R* = 100 voxels was generated, where Voronoi cells were confined.

Seeds placement was defined by the following parameters: *r*, radius from the sphere centre at which placing the seeds that would result in outer cells (*r*<*R*); *P*, the probability that a cell seed lies within the embryo's interior; cell seeds that satisfy the condition *P*, were constrained to a region bounded by two concentric spheres of radius *d* (inner) and *D* (outer), where (*d* < *D* < *r*). Additionally, a minimum intercellular distance *D_2* = 3 voxels was imposed using a Poisson‐disk criterion to ensure computational feasibility.

Under these constraints, most of the *N* seeds were positioned at radius *r*, spaced at least *D_2* apart from each other. A smaller fraction of seeds, determined by *P*, was randomly placed within the embryo interior, in the domain defined by *d* and *D*.

To choose realistic parameter values, we measured cell positions in all embryos of our dataset that contained inner cells (N = 15). We found that outer‐cell centroids were, on average, located at 69% ± 2% of the embryo's principal axis length (from sphere centre), while inner‐cell centroids were at 33% ± 13%. Based on these values, we set *r* = 70 and *d* = 20 voxels. The value of *D* was fixed at 60 voxels, slightly higher than the observed values, to account for the presence of cells that were not strictly classified as inner, as they remain in slight contact with the outer surface but have their centroid located toward the embryo's interior. The parameter *P* was set to 0.1, consistent with the observation that embryos typically contain one to two inner cells at 16‐cell stage (see Figure S9A [64, 71]).

Lloyd's algorithm (up to five iterations) was applied as defined in the previous model. We computed 100 simulations for each cell number. The resulting 3D Voronoi tessellations were used to extract quantitative information on cellular connectivity, including surface and full 3D neighbours, as well as on cellular morphology, such as exterior surface area, perimeter, and cell volume, following the methodology described in ref. [29].

#### 4.6.3 Analytical Voronoi on Spherical Surfaces

To quantify the frequency with which each *theoretical configuration* manifests on small biological shell surfaces, we implemented an analytical 3D Voronoi model on a spherical surface. This formulation allows rapid computation of cell areas, perimeters, and polygonal classes, as vertices are calculated directly in Euclidean space rather than in voxelised or meshed representations. On spheres, these quantities can be derived from established geometric formulas based on vertices positions. However, the model does not reproduce spheroidal shapes or quantify volumetric features such as apico‐basal intercalations (scutoids). The implementation was based on the MATLAB function VoronoiSphere.

For each simulation, a predefined number of seeds (*nSeeds*) were randomly distributed on the unit sphere and normalised to lie on its surface. Voronoi tessellations were computed to generate cell vertices, boundaries, and connectivity. Tessellations were iteratively relaxed using Lloyd's algorithm (up to five iterations).

For each Voronoi cell, the polygonal type (triangles to octagons), area and perimeter were calculated. Cell areas were determined using L'Huilier's formula for polygons on spherical surfaces, while perimeters were calculated as the sum of angular distances between consecutive vertices. Polygon types were categorised according to the number of vertices (from 3 to 8).

Across 100 000 realisations, configurations were collated to compute summary statistics for each unique polygonal arrangement (Tables S3 and S5). For each configuration, we quantified the mean and standard deviation of cell areas and perimeters, as well as a cellular *compactness descriptor* defined as $\sqrt{area}/perimeter$. This metric represents the mathematical inverse of the standard cell ‘*shape index*’, ($perimeter/\sqrt{area}$), proposed by [54]. In this form, higher *compactness descriptor* values correspond to greater cell compactness, which is consistent with classical shape‐characterisation metrics [55]. The mean and standard deviation of this *compactness descriptor*, alongside configuration frequencies, were recorded for each Lloyd iteration and exported to summary tables for downstream analyses (Tables S3 and S5). This framework provides a comprehensive baseline of polygonal configurations on spherical surfaces, facilitating comparison with biological tissues.

### 4.7 Gaussian Curvature Per Cell Patch

The Gaussian curvature associated with each cell patch was computed using a discrete differential geometry approach on a triangulated surface mesh [72]. A Delaunay triangulation [73] was applied to the vertices that define the spheroid surface, providing the local connectivity required for discrete curvature estimation. Gaussian curvature at each mesh vertex was then estimated using the angle‐deficit formulation [72, 74]. In this formulation, the Gaussian curvature at each vertex is estimated from the deviation of the sum of the interior angles of the triangles incident on that vertex from the 2π angle expected for a locally flat surface. The integrated Gaussian curvature of each cell patch was then obtained by summing the area‐weighted curvature contributions of all vertices within the patch.

For simplification, and to provide a more intuitive interpretation of local curvature, we also estimated the Gaussian curvature associated with each cell patch using Gauss–Bonnet's theorem [45]. According to Gauss–Bonnet's theorem, the total integrated Gaussian curvature of a spheroid surface is equal to 4π. Assuming that cell areas are approximately homogeneous across the spheroid surface, the Gaussian curvature associated with each cell patch can therefore be approximated as 4π/N, where N is the total number of cells. A comparison between this approximation and the discrete differential geometry approach described above is shown in Figure S1B,C.

### 4.8 Polygon‐Polygon Contact Matrices

Using the neighbourhood information of each cell in the tessellation, whether derived from real or simulated tissues, cells were first classified according to their number of neighbours (polygon class) ranging from triangles (three‐sided cells) to nonagons (nine‐sided cells). Neighbouring cell pairs were then grouped according to the polygon classes of the two cells (e.g., pentagon–pentagon, pentagon–hexagon, pentagon–heptagon, …), ensuring that each pair was considered only once. This procedure effectively constructs an adjacency matrix representing the polygon‐polygon contact network.

### 4.9 Statistical Analysis

Statistical analyses were performed using GraphPad (Prism).

#### 4.9.1 Data Binning

To quantify the polygonal distribution of Control cysts as a function of cell number, cysts were grouped into eight discrete bins according to their total cell count (Table S2). Each bin was established around a specific number, that we used to name it, and included cysts within a defined range. The bins were defined as follows: (15–25, N = 95 cysts) for the 20‐cell bin, (26–35, N = 74 cysts) for the 30‐cell bin, (36–45, N = 55 cysts) for the 40‐cell bin, (46–55, N = 76 cysts) for the 50‐cell bin, (56–65, N = 58 cysts) for the 60‐cell bin, (66–75, N = 39 cysts) for the 70‐cell bin, and (76–85, N = 24 cysts) for the 80‐cell bin. To ensure a sufficient number of samples for statistical analysis, a broader final bin settled around 100 cells value, was defined to include cysts with 86–125 cells (N = 38 cysts). This binning strategy enabled consistent statistical comparisons between experimental data and Voronoi‐based simulations across a broad range of cyst sizes. For cysts cultured in 2%FBS (Table S2), data were organised following the same approach considering four bins: 20‐cell (N = 142 cysts), 30‐cell (N = 75), 40‐cell (N = 30 cysts) and 50‐cell (N = 15 cysts). For ROCK‐inhibited cysts (Table S2), two bins were defined: 20‐cell (N = 43 cysts) and 30‐cell (N = 22 cysts).

#### 4.9.2 Global Comparison of Polygon Distributions Between Conditions

Control and 2%FBS polygon distributions were compared to each other, as well as against the predictions of the oblate spheroidal Voronoi model (Figures 2E and 3D and Figures S2B and S4G). Comparisons were performed using a paired Wilcoxon signed‐rank test to evaluate differences between experimental and simulated frequencies. Spearman's rank correlation coefficient was computed to assess the strength and direction of associations between predicted and observed distributions.

#### 4.9.3 Individual Comparison Between Conditions

To determine the appropriate statistical method, data distribution was first assessed using normality tests (Shapiro–Wilk). Normally distributed data with equal variances were analysed using a standard Student's *t*‐test or ordinary one‐way ANOVA. If data did not satisfy these conditions, non‐parametric tests (Mann–Whitney *U* test and Kruskal–Wallis test) were applied. To compare the overall polygon distributions between Control, 2%FBS and ROCK‐inhibited cysts at different bin numbers, Permutational Multivariate Analysis of Variance (PERMANOVA) was performed (Figures 3E,F and 4D; Figures S4E,F, S5B, and S6G). This test was also used to compare the distribution of Voronoi simulations with experimental data from mouse embryos, both for outer polygon distribution and total number of neighbours (Figure 6C and Figure S9B). In case of significant differences, a post‐hoc test was applied to compare each polygon class: Kruskal–Wallis test with Dunn's multiple comparison correction (Figure 6D and Figure S9C,D). Two way‐ANOVA with Sidak's multiple comparisons correction was used to analyse the effect of 2%FBS in cell proliferation and cysts formation efficiency across time (Figure S3A,B). For morphological features comparisons between 16‐ and 8‐cell MDCK cysts, two‐tailed Student's *t*‐test (Figure 4C and Figure S6B,D,F) or Mann–Whitney test (Figure S6A,C) were used. Multiple Mann–Whitney tests with Bonferroni correction were used in comparisons of number of cells and morphological features at different days of culture or bin number (Figure 3B and Figures S3C,D, S4A–D, and S5C–F).

## Author Contributions

L.M., C.G., and V.A. conducted the cell‐culture experiments, immunostaining, and imaging of MDCK‐II cysts in different conditions with help from A.M.P. J.A.A.‐S.R. carried out all the computational experiments, including tissue modelling, image segmentation, and image analysis. P.G.‐G. and J.G.‐G. contributed to the design of the computational models. L.M. curated the cell segmentation from confocal images of cysts and mouse embryos with help from E.L.‐F. K.M. provided the mouse embryo volumes and supported their analysis. L.M.E., J.A.A.‐S.R., V.A., and L.M. designed the figures. P.G.‐G., V.A., and L.M.E. conceived the study, supervised the experiments, and wrote the paper with input from all authors. All authors participated in the interpretation of the results, discussions, and development of the project.

## Conflicts of Interest

The authors declare no conflicts of interest.

## Supporting information




**Supporting File 1**: advs77487‐sup‐0001‐SuppMat.docx.

**Supporting File 2**: advs77487‐sup‐0002‐DataS1.xlsx.

**Supporting File 3**: advs77487‐sup‐0003‐DataS2.xlsx.

**Supporting File 4**: advs77487‐sup‐0004‐DataS3.xlsx.

**Supporting File 5**: advs77487‐sup‐0005‐DataS4.xlsx.

**Supporting File 6**: advs77487‐sup‐0006‐DataS5.xlsx.

## Data Availability

The data that support the findings of this study are openly available in GitHub at https://github.com/ComplexOrganizationOfLivingMatter/epithelialShellModelling/releases/tag/v1.0.0.
