# Supplementary material for: The Interplay of Curvature, Geometry, and Topology Shapes Tissue Organisation in Epithelial Shells
Source: Adv Sci (Weinh). 2026 Sep 28:e77487. Online ahead of print. doi: 10.1002/advs.77487 (PMC13617628; doi:10.1002/advs.77487)
Supplement: Supplementary file 1 [file ADVS-9999-e77487-s005.docx]

**Supporting Information for The interplay of curvature, geometry and topology shapes tissue organisation in epithelial shells**

Laura Morato^1,†^, Jesús A. Andrés-San Román^1,†^, Juan Garrido-García^2^, Carmen Gordillo-Vázquez^1^, Ana M. Palacios^1^, Elisabeth Lidueña-Fernández^1^, Katie McDole^3^, Pedro Gómez-Gálvez^1,4,^*, Valentina Annese^1,^*, Luis M. Escudero^1,4,^*.

**This PDF file includes:**

**Supplementary figures**:

figs. S1 to S9

**Other Supplementary Materials for this manuscript include the following:**

table S1. Polygon distribution data from Voronoi simulations, both sphere and spheroid models.

table S2. Experimental data from Control, 2%FBS and ROCK-treated cysts.

table S3. Theoretical and ranked configurations for 8- and 16-cells, including frequencies in cysts and corresponding Voronoi models for those within the ranking.

table S4. Polygon distribution data from mouse embryos and Voronoi embryo models (Voronoi 2 and Voronoi 6).

table S5. Ranked configurations for 8, 15 and 16 outer cells embryos and corresponding Voronoi models.

**fig. S1. The 3D Voronoi model reproduces the curvature effect in epithelial organisation. A)** Progressive relaxation of Voronoi tessellation on spherical surfaces with increasing Lloyd iterations (**Methods**). Six spheres are shown from left to right, beginning with a random configuration (Voronoi 1, no iterations) and followed by successive homogenisations of the distance between the seeds (Voronoi 2–6). **B)** The ratio of individual cell area to the total Voronoi surface area (left axis, grey) is plotted alongside the theoretical function of curvature per cell patch (4π/N; right axis, dark grey). The uniformity of cell areas is reflected in the dispersion around the function, suggesting limited variation in expected curvature. As the number of cells increases (X-axis), the ratio approaches zero, consistent with convergence toward planar behaviour. **C)** Mean Gaussian curvature per cell patch calculated for spheres and spheroid models of different cell numbers. While spheroidal variants exhibit a larger standard deviation than spheres due to their varying polar-to-equatorial geometry, the average curvature per cell patch remains extremely similar between geometries. Shaded areas represent the s.d. for spheres (dark green) and spheroids (light green). **D-E)** Average polygon distribution of the inner oblate spheroidal surface (D) and outer spherical surface (E) of 3D Voronoi models with increasing cell number (N=20 samples for each number of seeds). Simulations with fewer cells show a higher proportion of pentagons, whereas increasing the number of cells leads to convergence toward the planar epithelial distribution (data from (*3*)).

**fig. S2. The 3D Voronoi model reproduces the polygon distribution of epithelial shells. A)** Supporting image for **Fig. 2E** showing the frequency of each polygonal class on the outer surface for each sample (scatter plot). Triangles indicate the mean for each bin, with error bars showing the s.d. **B)** The Voronoi model predicts the average distribution of polygons on the inner surface of MDCK cysts at different sizes (see **Methods**). The dashed line is a visual guide connecting the mean values of each bin. Shaded areas represent the s.d. Coloured dots indicate the average values of each polygon class for Voronoi simulations, with error bars representing the corresponding s.d. There are no significant differences between both sets of data by Wilcoxon paired test. Spearman's correlation coefficient: 0.98. **C)** The ratio of individual cell area to total cyst surface area is shown for individual cells along the theoretical per-patch curvature function, analogous to **fig. S1B**. The narrow dispersion indicates nearly uniform cell-patch curvature, closely tracking the theoretical decay toward zero with increasing cell number.

**fig. S3. Reduction of FBS concentration affects MDCK cell proliferation and cyst development. A)** Growth curve of a 2D culture showing the number of viable cells (Y-axis) over time (X-axis) for Control and 2%FBS MDCK cells. A total of 4000 cells were cultured from the time of seeding (see **Methods**). Data represent mean ± s.d from three independent assays, each with three replicates.

* p<0.05; ** p<0.01 by two-way ANOVA with Sidak’s multiple comparisons correction. **B)** Quantification of cysts formation per assay at different culture time points for Control and 2%FBS MDCK cysts. Each condition and time point includes a minimum of three independent replicates. Cyst formation efficiency was similar across conditions, with no significant differences detected by two way-ANOVA with Sidak’s multiple comparisons. **C-D)** Comparison of cyst volume (C) and lumen volume (D) at three culture times (4, 7, and 10 days). In both panels, 2%FBS cysts exhibited significantly smaller volumes compared to Control cysts. Each dot corresponds to one cyst, and bars indicate mean ± s.d. ***p < 0.001 by Mann–Whitney tests with Bonferroni multiple comparisons correction.

**fig. S4. 2%FBS and Control cysts differ morphologically but share the outer-surface polygon distribution. A–D)** Comparison of cyst volume (A), lumen volume (B), cell height (C), and cell volume (D) for Control cysts and 2%FBS cysts. The X-axis represents cell number, grouped into four bins (see **Methods**). **p < 0.01; ***p < 0.001 by Mann-Whitney tests with Bonferroni multiple comparisons correction. **E-F)** Polygon distributions of Control and 2%FBS cysts for 20-cell bin [15–25 cells], (E) and 40-cell bin [36-45 cells], (F). Statistical significance of the overall distributions was evaluated by PERMANOVA (p<0.001 for 20-cell bin) followed by post-hoc Mann-Whitney tests with Bonferroni multiple comparisons correction. *p<0.05; **p<0.01. When compared individually for each polygon class, small differences arise at the 20-cell bin, probably due to a higher frequency of 16-cell cysts in controls. **G)** Outer surface polygon distribution in 2%FBS cysts within the 20–50 cell range. Data from 2%FBS cysts are presented as squares representing the mean, with error bars indicating the s.d. Data for Control cysts are shown as a dotted line connecting the mean of each bin, with a shaded area representing the s.d. There are no significant differences between both sets of data when compared globally by Wilcoxon paired test. Spearman's correlation coefficient: R: 0.97.

**fig. S5. ROCK inhibition during cystogenesis does not affect outer surface organisation. A)** Confocal sections of MDCK Control cyst (left, dark red) and treated with ROCK inhibitor (Y-27632, 20μM) from seeding (right, turquoise). In the top images, cysts were immunolabeled by β1-integrin and pMLC primary antibody in addition to phalloidin staining. Bottom images, note that in untreated cysts, pMLC (grey) is concentrated at the outer surface. In the presence of the ROCK inhibitor, this pattern disappears. Scale bar, 10μm. **B)** Average polygon distribution at the outer surface of both Control and ROCK-inhibited cysts for the 20-cell bin [15–25 cells] (left) and the 30-cell bin [26–35 cells] (right). Data are shown as mean + s.d. There are no significant differences between conditions for both groups of data by PERMANOVA. **C-F)** Comparison of cyst volume (C), lumen volume (D), cell height (E) and cell volume (F). Each dot represents a single cyst, and bars indicate the mean ± s.d. *p < 0.05 by Mann-Whitney tests with Bonferroni multiple comparisons correction.

**fig. S6. 2%FBS and Control cysts differ in shape but share inner-surface polygon distributions at 8-cell and 16-cell samples. A-B)** Comparison of lumen volume (A) and cell volume (B) for Control cysts and 2%FBS cysts of 16 cells. ***p<0.001 by Mann-Whitney test, except in B) where two-tailed Student's t-test was used. **C-F)** Comparison of cyst volume (C), lumen volume (D), cell volume (E) and cell height (F) for Control and 2%FBS cysts of 8 cells. Each dot represents a single cyst, and bars indicate the mean ± s.d. **p<0.01; ***p<0.001 by two-tailed Student's t-test, except in (C) where Mann-Whitney test was used. **G)** Polygon distribution of Control and 2%FBS cysts for 8 and 16 cells at the inner surface. Data are shown as mean + s.d. There are no significant differences in the 16-cell group by PERMANOVA. The statistical comparison was omitted for 8 cells (zero variance).

**fig. S7: Frequency of configurations and adjacency matrices for higher-ranked configurations at 16 cells.** **A)** Frequency of each configuration per 3D Voronoi model geometry: sphere, oblate spheroid and prolate spheroid. The frequency of high-ranked configurations is similar between the three geometries. **(B–G)** Bubble plots showing the frequency of each polygon-polygon contact, where bubble size is proportional to the number of contacts of that type. Each row displays a distinct adjacency matrix associated with the same configuration. The occurrence frequency of each matrix is indicated for both Voronoi spheroids (grey) and Control cysts (red).

**fig. S8. Configurations on the inner and outer surfaces of MDCK cysts. A)** Supporting image for **Fig. 5F**, presenting analogous data from 2%FBS samples. Frequencies are shown for (from top to bottom): 2%FBS cysts without scutoids, where the inner and outer surfaces have identical configurations (2%FBS no scutoids); the inner surface of 2%FBS cysts with scutoids (2%FBS scu. inner) and the outer surface of 2%FBS cysts with scutoids (2%FBS scu. outer). **B)** Heatmap showing all the configurations that appear in cyst with scutoids. For each configuration, the numerical rank position is indicated. “Out” represents the only configuration that does not appear in the rank (1-5-5-2-0-3, represented in D). Top: Comparison of the frequency of configurations in the inner surface of the cysts with only one apico-basal intercalation (1 A-B I., N=22) and more than one (>1 A-B I., N=10). Bottom: Same comparison for the outer surface. The presence of more than one apico-basal intercalation correlates with a major presence of low-ranked configurations in the inner surface and high-ranked configurations in the outer surface. **C)** Heatmap of apico-basal polygonal intercalations in scutoids from Control cysts. The heatmap depicts the frequency of each apical-to-basal polygon combination, with each entry representing scutoids linking a polygon with a given number of neighbours on the inner (apical) surface to a polygon with a given number of neighbours on the outer (basal) surface through apico-basal intercalations. The three most common transitions from inner to outer are: four to five, five to six and six to five neighbours. **D)** Segmentation of a Control cyst with one cell dividing. The outer surface presents a configuration with a very low-ranking position (rank 74). The inner surface configuration is theoretically possible but does not appear on the ranking of configurations after 100,000 realisations.

**fig. S9. Voronoi 2 closely reproduces the outer organisation of 8-cell-stage embryos. A)** In 16-cell embryos, the Voronoi 2 model (dark grey) reproduces the distribution of surface-contacting cells more closely than models with additional iterations (Voronoi 6, light grey). The X-axis shows the number of cells in contact with the exterior, and the Y-axis the percentage of embryos exhibiting each outer-cell count. **B)** Segmented image of a 16-cell embryo with a cell that appears twice on the outer surface. Left: the cell of interest is coloured in dark red. Right: Same embryo with cells coloured depending on their number of cell contacts. Below the 3D representation, the configuration is shown with the polygon colour-code. **C-D)** Average polygon distribution on the outer surface (C) and the average total number of neighbours (D) for 8-cell real embryos (magenta) compared with 8-cell Voronoi simulations after one and five Lloyd iterations (Voronoi 2 and Voronoi 6, respectively). Statistical significance of the overall distributions was evaluated by PERMANOVA (p<0.01, for comparison of mouse embryos with Voronoi 6 at outer surface and p<0.001 for total number of neighbours, with both Voronoi 2 and Voronoi 6), followed by post-hoc Kruskal-Wallis tests with Dunn's multiple comparisons correction to identify differences between polygon classes. *p<0.05; **p<0.01; ***p<0.001. **E)** 3D reconstructions of 8-cell embryos with schematic representations of their polygonal configurations. Two embryos are shown, corresponding to the most frequent configurations. Left: four squares and four pentagons (10/23 cases). Right: one triangle, three squares, three pentagons, and one hexagon (7/23 cases).
